# Memory-Based or Experience-Based? Subject-Object Asymmetry in Mandarin Relative Clause Processing from the Aging Perspective

**DOI:** 10.3390/bs16050646

**Published:** 2026-04-25

**Authors:** Xinmiao Liu, Jiani Shi, Shengqi Wu

**Affiliations:** 1 National Research Centre for Foreign Language Education, Beijing Foreign Studies University, Beijing 100089, China; 2 School of English for Specific Purposes, Beijing Foreign Studies University, Beijing 100089, China

**Keywords:** relative clauses, sentence processing, aging, subject-object asymmetry, Mandarin Chinese

## Abstract

The processing difficulty of subject relative clauses (SRCs) and object relative clauses (ORCs) in Mandarin Chinese has been a controversial issue in psycholinguistics. Memory-based accounts and experience-based accounts make contrastive predictions regarding the processing asymmetry. Given that older adults tend to have lower memory and richer language experience, the processing of relative clauses in older adults can reveal which theoretical account offers a more adequate explanation of sentence comprehension. The present study compared the processing of Mandarin SRCs and ORCs in older and younger adults using a self-paced reading paradigm. The results revealed that both groups showed lower accuracy for SRCs than ORCs, but this effect was larger in older adults. These age-related differences cannot be attributed to different strategic trade-off mechanisms and task experience. During online processing, older adults performed more slowly than younger adults, but no significant interaction was found between age and RC type. These findings suggest that aging affects sentence comprehension at the post-interpretive and decision processes, while online syntactic processing remains relatively preserved but globally slowed. The findings are largely consistent with memory-based accounts, indicating that age-related changes in sentence comprehension may be attributed to memory decline in older adults.

## 1. Introduction

Relative clauses (RCs) are the clauses modifying nouns or noun phrases in sentences. Based on the syntactic role of the head noun within the clause, RCs can be divided into subject relative clauses (SRCs) and object relative clauses (ORCs). As shown in the examples below, sentence (1a) is an SRC, and sentence (1b) is an ORC.
(1)a.The man [who__ attacked the driver] ran away. (SRC)
b.The man [who the driver attacked __] ran away. (ORC)

The RC is indicated by square brackets, and the gap position left by the extracted constituent is indicated by an underscore. The extracted constituent “the man” serves as the subject of the main clause in both sentences, which means that both RCs are subject-modifying. The difference is that “the man” is the grammatical subject of the RC in sentence (1a) and the object in sentence (1b). RC, as a structure involving non-local dependencies and high cognitive load, has long been used to test competing accounts of sentence processing. In particular, the processing asymmetry between SRCs and ORCs has been debated across languages. In head-initial languages such as English and Italian, many studies have reported that ORCs are more difficult to process than SRCs (Guasti et al., 2018; King & Just, 1991; Traxler et al., 2002). RC processing is therefore a useful domain for exploring how structural complexity, memory and linguistic experience interact during comprehension.

Two broad classes of accounts have been proposed to explain RC processing difficulty: memory-based accounts and experience-based accounts (Gibson & Wu, 2013). A representative theory of memory-based accounts is the Dependency Locality Theory (DLT; Gibson, 1998, 2000). This theory posits two main costs in comprehension: integration cost and storage cost. Integration cost arises at the point where a syntactic dependency is completed and is proportional to the linear distance between the head and its dependent. Storage cost reflects the memory resources required to maintain predictions about upcoming syntactic structure and increases with the number of unresolved dependencies. Under this view, structures involving longer or more demanding dependencies should be more difficult to process. A structure-based account relevant to RC processing is the Structural Distance Hypothesis (O’Grady, 1997; O’Grady et al., 2003). This account holds that processing difficulty increases with the structural distance between syntactically related constituents. The more deeply embedded the dependency is, the greater the processing burden.

Experience-based accounts (Mitchell et al., 1995) propose that sentence processing is based on experience, and the processing difficulty of sentences is determined by the probabilistic knowledge derived from linguistic experience. Such accounts include the frequency-based theory (Reali & Christiansen, 2007), the surprisal theory (Hale, 2001), and the expectation-based account (Levy, 2008). These theories make predictions of processing difficulty according to the frequency of distribution and probability. For example, Reali and Christiansen (2007) demonstrated that RC processing difficulty can be predicted from distributional frequencies in the input. More generally, probabilistic parsing models define processing difficulty in terms of surprisal, which is the negative log probability of an input given its context (Hale, 2001; Levy, 2008).

Mandarin Chinese provides an informative testing ground for these competing accounts. Unlike English, Chinese is a head-final language with a canonical word order of SVO (e.g., Dryer, 1992; C.-J. C. Lin, 2008; Wu et al., 2012). In Chinese, the RC precedes the head noun and is marked by the relativizer *de*. This typological property changes the linear order of the dependency. The examples in (2) illustrate the two types of Chinese RCs.
2.(a) 批评学生的老师很生气。
[__piping xuesheng de] laoshi hen shengqi. (SRC)
 criticize student de teacher very angry.
“The teacher who criticized the student was very angry.”
(b) 老师批评的学生很生气。
[laoshi piping __de] xuesheng hen shengqi. (ORC)
 teacher criticize de student very angry.
 “The student whom the teacher criticized was very angry.”

Although memory- and experience-based accounts can predict the processing advantage of English SRCs, their predictions for Chinese RCs are highly divided. In the case of RCs, DLT predicts processing asymmetries based on the distance between the filler and the gap. As shown in the example above, Chinese SRCs like (2a) involve three syntactic heads (*piping*, *xuesheng*, *de*) between the head noun “xuesheng” and its syntactic position within the clause, leading to higher storage costs. ORCs like (2b) have only one syntactic head “de” between the head and the syntactic position, resulting in lower memory demands. As the linear distance between the filler and the gap is longer in SRCs, it is more resource-consuming to complete the syntactic integration. Therefore, the theory predicts that SRCs are more difficult to process than ORCs (Gibson, 1998, 2000). In contrast, the Structural Distance Hypothesis predicts an SRC advantage over ORCs. Under this view, the dependency involved in an SRC is structurally simpler than that in an ORC, and thus ORCs should be more difficult to process.

Evidence from corpus studies consistently demonstrated that Chinese SRCs were more frequent than ORCs (Pu, 2007; Vasishth et al., 2013). Accordingly, the experience-based accounts predict that Chinese ORCs are more difficult to process than SRCs. This prediction was confirmed by C.-J. C. Lin and Bever (2006). Y. Hsiao and MacDonald (2013) investigated Mandarin Chinese RC processing with a connectionist approach, combining corpus analysis with a simple recurrent network. The model was then tested on 16 RC subtypes, and its grammatical prediction error was compared with patterns in previous reading-time studies. They found that processing difficulty depends on the interaction of RC type, the modifying position of RCs, and the animacy of nouns. In this view, Mandarin RC processing is shaped less by a working memory burden than by probabilistic expectations, structural competition, and ambiguity resolution based on linguistic experience. Previous studies suggest that experience-based approaches predict an ORC disadvantage in Mandarin, but they differ in how that prediction is derived and in how much weight is given to overall frequency and other distributional factors. An additional complication in Mandarin RC processing is that temporary ambiguity may arise during incremental parsing. The relativizer *de* can be temporally ambiguous in ORCs (Zhou et al., 2018), because it often signals possession. This ambiguity persists until readers encounter the main clause verb and is likely to make ORCs more difficult to process. As a result, observed processing differences between SRCs and ORCs may reflect not only RC type per se but also the interaction between structural integration and ambiguity resolution.

Consistent with these competing predictions, previous findings on Mandarin RC processing have been highly mixed. Some studies have found that Chinese SRCs are more difficult to process (e.g., Chen et al., 2008; F. Hsiao & Gibson, 2003; Y. Lin & Garnsey, 2010; Qiao et al., 2012; Gibson & Wu, 2013; Kong, 2024). Other studies suggest that ORCs are more difficult to comprehend (e.g., C.-J. C. Lin & Bever, 2006). Still others report that there is no RC processing asymmetry in Chinese (e.g., Cheng, 2023). These inconsistent findings suggest that Mandarin RC processing cannot be reduced to a single factor. Instead, the RC asymmetry depends on how memory demand, structural complexity, probabilistic expectation, and temporary ambiguity interact under different task and design conditions. Therefore, Mandarin RCs provide a useful context for further testing competing accounts of sentence processing.

Most previous experimental studies investigating the processing of Chinese RCs have focused on young adults or children (e.g., Diessel & Tomasello, 2005; Fluck, 1978; Xu et al., 2019), and the aging population has received less attention. Research on sentence processing in aging has shown that older adults often differ from younger adults in cognitive resources and comprehension performance. Older adults tend to have lower working memory spans and slower processing speed, and they perform less accurately in comprehending syntactically complex structures (Caplan et al., 2011; G. S. Waters & Caplan, 2001). However, previous studies have also suggested that age-related differences do not necessarily reflect a general loss of syntactic ability. For example, G. S. Waters and Caplan (2001) found that age differences were greater in offline measures than in online measures, and complex sentences were more difficult to comprehend as they placed heavier demands on domain-general resources (Liu & Wu, 2026). Other studies have suggested that aging may also affect how processing effort is allocated during comprehension. Stine-Morrow et al. (2006) proposed that language processing in older adults is shaped by reduced processing capacity, increased knowledge, and changes in resource allocation. DeDe (2014) further suggested that older adults may rely more on probabilistic cues during sentence comprehension. Such findings are directly relevant to the present study, because RC processing places demands on both syntactic computation and memory. Importantly, Liu et al. (2019) found that older Mandarin speakers were slower and less accurate than younger speakers in the processing of RCs and interpreted this pattern as more consistent with limitations in working memory. Against this background, comparing younger and older adults in Mandarin RC processing may help clarify the underlying reasons of age differences in comprehension.

Previous research has found that ORCs are more difficult for older adults to process (Baum, 1993; G. S. Waters & Caplan, 2001; Kemper et al., 2004; Kemper & Liu, 2007; DeDe, 2015). However, most of these studies have focused on head-initial languages such as English, and the findings require further validation from studies of head-final languages. Examining the processing of Chinese RCs could provide cross-linguistic evidence regarding the RC asymmetry among older adults. Due to the typical characteristics of older adults, such as working memory decline and extensive language experience (Baltes et al., 1999; Bopp & Verhaeghen, 2005), this group provides a valuable opportunity to distinguish between competing accounts of processing difficulties of RCs. The Dependency Locality Theory suggests that the storage and integration costs of Chinese SRCs are higher than those of ORCs (Gibson, 1998, 2000). With increasing age and working memory decline, older adults are more sensitive to the processing costs of syntactic structures (Payne et al., 2014), and the effect of the difference in processing costs might be amplified in older adults. As a result, the ORC processing advantage is expected to be stronger in older adults. In contrast, experience-based accounts suggest that the processing difficulty of syntactic structures is related to the statistical probability at the syntactic, semantic, and pragmatic levels. Frequently occurring inputs in the environment will strengthen our memory of the relevant structures. The higher the frequency of a linguistic structure, the more proficient an individual is in processing that structure, and the lower the difficulty. Previous corpus-based studies have reported that Mandarin SRCs are more frequent than ORCs (e.g., Pu, 2007; Vasishth et al., 2013), and thus SRCs are predicted to be easier to process than ORCs. Extending this logic to aging, one may expect that older adults, because of their greater cumulative exposure to language, would be more sensitive to this distributional difference. The difference in processing difficulty between the two types of RCs is expected to be greater in older adults. That is, the SRC processing advantage might be more pronounced in older adults. Thus, the two accounts diverge particularly sharply in their predictions for older adults, making this population especially useful for distinguishing them.

In this study, a self-paced reading experiment was administered among a group of older and younger adults to investigate the processing of Chinese SRCs and ORCs. Our research question is which type of RC is more difficult to process in older adults. If memory-based accounts are correct, older adults should show greater difficulty with SRCs, the more resource-demanding RC type, and an RC effect is expected to be greater in older adults than in younger adults. If experience-based accounts play the dominant role, older adults’ richer linguistic experience may amplify sensitivity to distributional regularities, and thus they should encounter greater difficulty for ORCs than for SRCs. By examining both online reading times and comprehension accuracy, the present study aims to clarify the nature of age-related differences in Mandarin RC processing and to evaluate the explanatory power of the two competing accounts. The findings can help us re-examine existing sentence processing theories from the perspective of aging and further our understanding of how language ability changes across the life span.

## 2. Materials and Methods

### 2.1. Participants

A total of 64 people participated in this experiment, including 35 elderly adults in the experimental group and 29 young adults as the control group. The average age of the younger group was 20 years (SD = 1.65, range = 18–26), and the average age of the older group was 67.2 years (SD = 5.98, range = 60–82). There was no significant difference in years of education between the two age groups, *t* = −0.19, *p* = 0.852. All participants were native speakers of Mandarin Chinese. Some participants reported previous experience learning an additional language such as English, Russian, or Japanese, but these languages were not used regularly in daily life, and Mandarin Chinese was the dominant language for all participants. Because such language experience was limited and heterogeneous, it was not analyzed in the present study. The participants were right-handed and had normal or corrected visual acuity. Prior to the experiment, the elderly participants underwent cognitive impairment screening using the Chinese version of the Mini-Mental State Examination (CMMSE, Katzman et al., 1988), and all participants showed no cognitive impairment. The young adults had higher working memory scores (M = 6.38) than the old adults (M = 5.00). A *t*-test showed that this difference was significant (*p* < 0.001). All participants provided informed consent for their participation, and received compensation after the experiment.

### 2.2. Materials

The experimental materials consisted of two types of Mandarin RCs: SRCs and ORCs. The two types of sentences differed only in the syntactic position of the RCs and the grammatical role of the extracted elements. Each sentence contained a single embedded RC. Both the subjects and objects of the RCs were animate and prototypical nouns. This allowed us to control for the confound of lexical animacy on sentence processing. A total of 30 sets of experimental sentences were used in this study. They were partly adapted from Liu et al. (2019)’s study. Each set contained an SRC and an ORC. Within each set, the lexical items were identical across conditions, and all sentences were controlled for length and segmented into six regions for analysis. The aspect marker *-le* following the main verb was treated as a tense or aspect marker and was grouped into the same region as the verb. In addition to animacy, temporo-aspectual cueing was also controlled across conditions. Within each item set, the predicate had the same aspectual marker. These 30 sets were then distributed into two experimental lists. List 1 contained SRCs from the first 15 sets and ORCs from the remaining 15, while list 2 contained the inverse. Consequently, each list consisted of 30 experimental items, including 15 SRCs and 15 ORCs. This ensured that each participant saw only one version of each sentence and an equal number of both conditions. An additional 30 sentences were selected as fillers. Fillers were distractor sentences that prevented participants from developing strategies. They helped to make the critical items less salient, encouraging more natural processing of experimental sentences. The fillers used in this study are of various structures and irrelevant to the structures of the experimental stimuli. The sentences were randomized before being presented to participants. Each sentence was followed by a question that probed participants’ comprehension. Sample experimental sentences are shown below. The vertical bar represents the boundary of segments in the sentence.
(1)Subject relative clause (SRC)
认识|老师|的|校长|见到了|学生
renshi | laoshi | de | xiaozhang | jiandao-le | xuesheng
know teacher de principal see-ASP student
“The principal who knows the teacher met the student.”(2)Object relative clause (ORC)
老师|认识|的|校长|见到了|学生
laoshi | renshi | de | xiaozhang | jiandao-le | xuesheng
teacher know de principal see-ASP student
“The principal that the teacher knows met the student.”
Question: Xiaozhang renshi laoshi ma? (“Does the principal know the teacher?”)

To ensure the acceptability of the experimental materials, all sentences were validated using a questionnaire. 98 adults across different age groups (M = 38.9; range: 18–70) participated in the norming test and rated the acceptability of all sentences on a 5-point Likert scale ranging from 1 (less acceptable) to 5 (most acceptable). Different age groups were included to ensure that the materials were acceptable across a broader adult population rather than only in one age group. These participants did not take part in the main experiment. The rating data were analyzed using a linear mixed-effects model. RC type was entered as a fixed effect, with random intercepts for subjects and items. The analysis showed that the effect of RC type was not significant, *β* = 0.13, *SE* = 0.10, *t* = 1.31, *p* = 0.194. This indicates that there was no significant difference in acceptability ratings between the two types of sentences.

### 2.3. Procedure

A self-paced reading task was implemented using moving window technology. The sentences were presented on the screen segment by segment. The experiment was conducted using E-Prime 2.0 (Schneider et al., 2012). All experimental materials were presented to participants on a computer monitor. The experiment consisted of two parts: a practice task and the main experiment. Participants first completed the practice task with eight practice trials. They were allowed to practice repeatedly until they understood the procedures. Sentences were first presented in underlined form. When a participant pressed a response key, a fixation point appeared on the screen, indicating the position where the sentence would appear. Each time a participant pressed the response key, a word was presented, and the previous word was masked by an underline. After the sentences were presented, a probing question showed up. Participants responded to the question by pressing one of two designated keys corresponding to “yes” and “no”. If no response was detected in ten seconds, the next trial started automatically. A 10 s response window was used to prevent excessively long pauses between trials and to maintain a consistent pace throughout the experiment. Responses not made within this time window were treated as missing values. No feedback was provided. After the reading task, participants’ working memory was assessed using the backward digit span task. The entire experiment took about 30 min.

### 2.4. Data Analysis

Data were analyzed using the packages *lme4* and *lmerTest* (Bates et al., 2015; Kuznetsova et al., 2017) implemented in R (version 4.4.2; R Core Team, 2024). Reading times were analyzed using linear mixed-effects models, whereas response accuracy was analyzed using generalized mixed-effects models. Random intercepts for subjects and items were included in all models. Reaction times were log-transformed before analysis. Trials with missing values were excluded. For the RT analyses, we focused on two regions of interest: the head noun and the main verb. The head noun here refers to the noun following the relativizer *de*, and it was defined as the critical region because it is the point at which the RC is expected to place integration costs on the parser. The main verb was analyzed as a spill-over region, since processing effects in self-paced reading often extend to the segment immediately after the critical region. In addition, extreme outliers were removed based on model residuals exceeding three standard deviations. To examine potential speed-accuracy trade-off effects, models were fitted with response times as a predictor of comprehension accuracy. We also performed a growth curve analysis on reading times across all regions to examine whether younger and older adults differed in their overall RT trajectories across the sentence. Statistical significance of fixed effects was assessed using Wald tests for generalized models and Satterthwaite-approximated *t*-tests for linear models. Post hoc comparisons were conducted using the *emmeans* package (Lenth, 2025), and all significance tests were evaluated at a level of 0.05.

## 3. Results

We first analyzed comprehension accuracy, including participants’ overall performance, speed-accuracy trade-off patterns, and potential effects of learning. Next, we examined response times to the comprehension questions as an additional measure of processing effort. Finally, we analyzed the online reading time data in critical and spillover regions. A growth curve analysis of reading times was also performed to explore the pattern of change across sentence regions in different age groups.

### 3.1. Comprehension Accuracy

**Figure 1:**
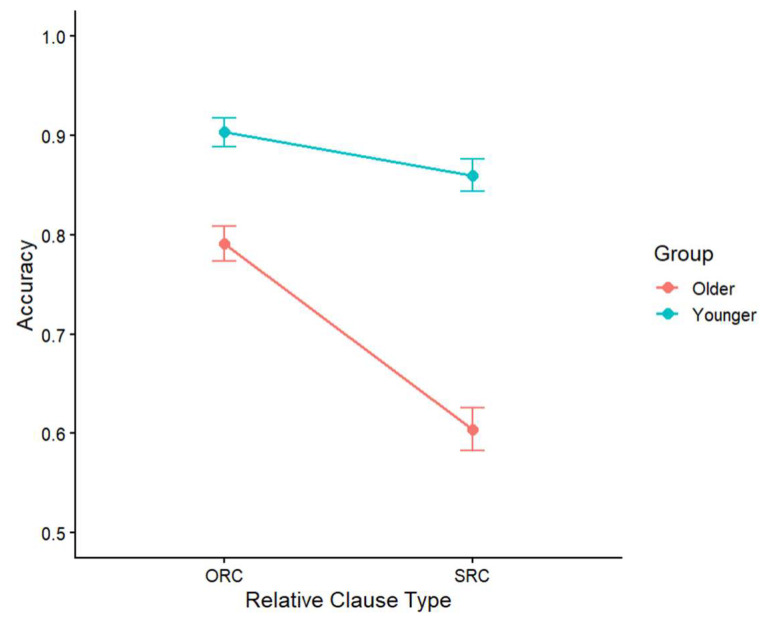
Mean accuracy for ORCs and SRCs in the young and older groups.

Accuracy data were analyzed using a generalized mixed-effects model with RC type (ORC vs. SRC), age group (young vs. older), and their interaction as fixed effects and random intercepts for subjects and items. The model was: acc ~ group * type + (1|subject) + (1|item), data = data, family = binomial, control = glmerControl(optimizer = “bobyqa”). The mean accuracy rate for the two groups is shown in Figure 1. The analysis revealed significant main effects of RC type. ORCs were responded to more accurately than SRCs (*β* = −0.99, *SE* = 0.15, *z* = −6.79, *p* < 0.05). The effect of age group was also significant (*β* = 1.06, *SE* = 0.28, *z* = 3.82, *p* < 0.05). The young group performed more accurately than the older group. The interaction effect between RC type and group was significant (*β* = 0.54, *SE* = 0.27, *z* = 2.02, *p* < 0.05), indicating that the effect of RC type differed between the two groups. Simple effects analyses showed that in the older group, accuracy was higher for ORCs than for SRCs (*p* < 0.001). In the young group, the difference between ORCs and SRCs was smaller but still significant (*p* < 0.05). Although both groups showed an SRC disadvantage, this effect was significantly larger in older adults, suggesting that aging increases processing difficulty for SRCs.

**Figure 2:**
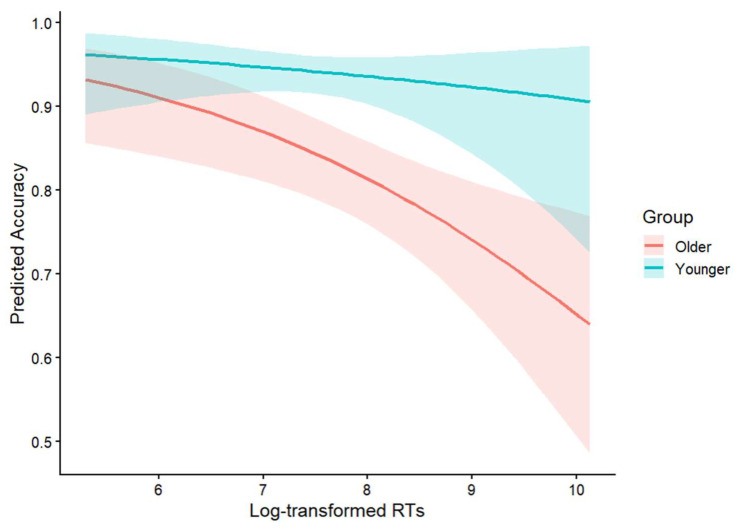
Speed-accuracy relationships in young and older adults.

Given that older adults may adopt more conservative strategies by trading speed for accuracy, it is crucial to explore whether age-related differences in accuracy reflect processing limitations or merely strategic adjustments. To address this issue, we conducted a speed-accuracy trade-off analysis. Specifically, we fitted a generalized mixed-effects model predicting accuracy from log-transformed response times, group, and their interaction, with sentence type as a control variable. The speed-accuracy trade-off patterns for the two groups are visually depicted in Figure 2. The analysis revealed a significant main effect of reaction times (*β* = −0.42, *SE* = 0.14, *z* = −3.02, *p* < 0.05), indicating that longer response times were associated with lower accuracy. This pattern reflects greater processing difficulty in more difficult trials, which tended to elicit both slower responses and lower accuracy. Although the figure suggests a steeper speed-accuracy relationship in the older group, the interaction between reaction times and group was not significant (*β* = 0.22, *SE* = 0.27, *z* = 0.83, *p* = 0.41). This indicates that the speed-accuracy relationship did not differ between young adults and older adults. Although both groups showed a significant relationship between reaction times and accuracy, aging did not modulate the strength of this relationship. The findings suggest that the group difference in accuracy cannot be explained in terms of different trade-off strategies.

Age-related differences in performance may reflect learning, adaptation, or fatigue over the course of the experiment. Given that older adults may differ from younger adults in their ability to adapt to task demands or maintain performance over time, it is important to determine whether observed group differences in accuracy reflect stable processing differences or dynamic changes across the experiment. To address this issue, accuracy was analyzed using a generalized linear mixed-effects model with trial order as a predictor, together with group and RC type as fixed effects. By-subject random intercepts were included in the model. The model revealed a significant main effect of group (*β* = 1.28, *SE* = 0.24, *z* = 5.27, *p* < 0.001), indicating higher accuracy in younger adults than in older adults. A significant main effect of clause type was also observed (*β* = −0.91, *SE* = 0.14, *z* = −6.46, *p* < 0.001), with lower accuracy for SRCs than for ORCs. The main effect of trial was not significant (*β* = 0.06, *SE* = 0.08, *z* = 0.81, *p* = 0.42). The interaction between trial and group approached statistical significance (*β* = −0.30, *SE* = 0.17, *z* = −1.78, *p* = 0.074), indicating a trend for the effect of trial order on accuracy to differ between younger and older adults. However, this effect did not reach significance, and therefore should be interpreted with caution. To further examine the source of this trend, follow-up analyses were conducted separately for each group. Trial order did not significantly predict accuracy in older adults (*β* = 0.08, *SE* = 0.08, *z* = 0.96, *p* = 0.338), and the younger group (*β* = 0.02, *SE* = 0.21, *z* = 0.07, *p* = 0.942). These results indicate that neither group showed reliable learning or adaptation effects across trials. This finding suggests that group differences in accuracy are unlikely to be driven by learning effect or fatigue over time.

### 3.2. Response Times in Comprehension Questions

**Figure 3:**
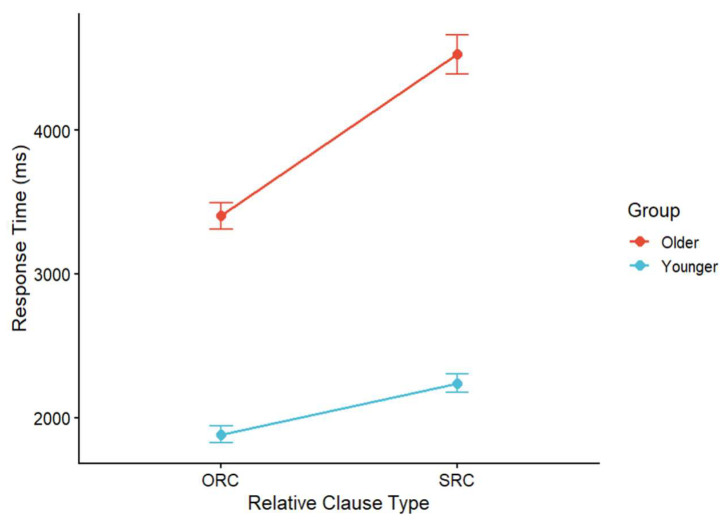
Mean response times for comprehension questions by age group.

Response times in the comprehension questions offer a complementary indicator of processing difficulty, reflecting the cognitive efforts during the decision and integration stage. It provides a more sensitive measure to detect subtle group differences in sentence comprehension. Therefore, we also analyzed the group differences in response times in comprehension questions. The data were log-transformed prior to analysis. Trials with missing values were removed, and extreme residuals exceeding three standard deviations were excluded based on a mixed-effects model. Mean response times by RC type and age group are shown in Figure 3.

A linear mixed-effects model was fitted to the log-transformed response times with group and RC type as fixed effects and random intercepts for subjects and items. The model revealed a significant main effect of group (*β* = −0.56, *SE* = 0.08, *t* = −6.77, *p* < 0.05), indicating that younger adults responded faster than older adults. A significant main effect of clause type was also observed (*β* = 0.31, *SE* = 0.03, *t* = 9.04, *p* < 0.05), with longer reaction times for SRCs than for ORCs. The interaction between group and clause type was significant (*β* = −0.12, *SE* = 0.05, *t* = −2.56, *p* < 0.05). Specifically, the difference between SRCs and ORCs was larger in older adults than in younger adults, indicating that older adults showed a higher processing cost for SRCs than ORCs. This pattern was largely consistent with the results of accuracy analysis.

### 3.3. Reading Time Analysis

**Figure 4:**
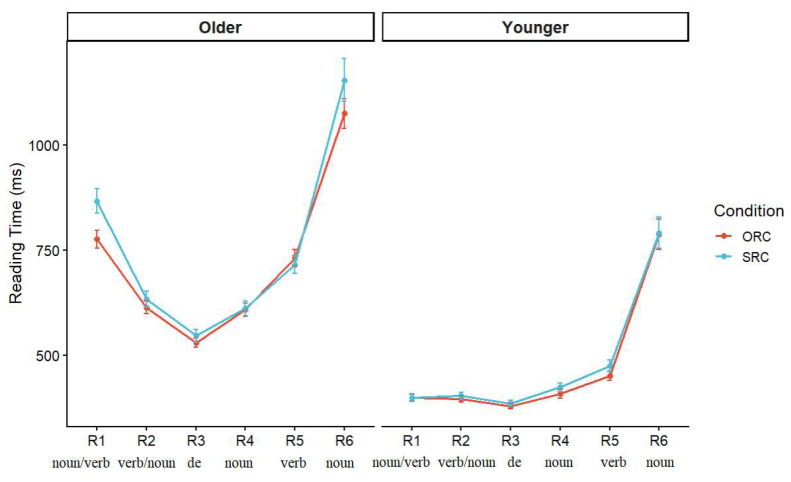
Mean RTs across sentence regions for older and younger adults (ms).

Next, we analyzed the reading times (RTs) in the critical regions of interest, the head noun of the RC and spillover region (main verb), to understand the online processing of RCs. For RT analyses, only trials with correct responses were included. This resulted in the exclusion of 419 out of 1920 trials, leaving 1501 trials for analysis. No participants were excluded from analysis. Prior to statistical analysis, outliers exceeding three standard deviations from the mean were removed for each region, resulting in the exclusion of 2.18% of the data in the older group and 1.96% in the younger group. The three-standard-deviation criterion is a conventional trimming procedure in RT research. Figure 4 shows the mean RTs for each segment in the two age groups. Mean reading times at each region are summarized in Table 1, and the results of statistical analysis were presented in Table A1 in Appendix A.

Reading times at head nouns were analyzed using a linear mixed-effects model with age group and RC type as fixed effects and random intercepts for subjects and items. The results revealed a significant main effect of group, indicating that younger adults responded significantly faster than older adults (*β* = −0.39, *SE* = 0.08, *t* = −4.82, *p* < 0.05). In contrast, neither the main effect of RC type (*β* = 0.001, *SE* = 0.02, *t* = 0.04, *p* = 0.97) nor its interaction with group reached significance (*β* = 0.02, *SE* = 0.04, *t* = 0.67, *p* = 0.50). Although older adults performed more slowly at the head noun, there was no reliable difference between SRCs and ORCs structures at this position in either group. RTs at the spillover region were also analyzed using a linear mixed-effects model. We found a significant main effect of group. Younger adults responded significantly faster than older adults (*β* = −0.45, *SE* = 0.08, *t* = −5.48, *p* < 0.05). The main effect of RC type was not significant (*β* = −0.01, *SE* = 0.03, *t* = −0.25, *p* = 0.80), nor was the interaction between group and type (*β* = 0.02, *SE* = 0.04, *t* = 0.63, *p* = 0.53). These results suggest that there was no difference in processing difficulty between the two types of sentences in either group at the spillover region. In the spill-over region, the analysis revealed a significant main effect of group (*p* < 0.01). Younger adults had shorter reading times than older adults. However, there was no significant main effect of RC type (*p* = 0.398), and no significant interaction between group and RC type (*p* = 0.359). No reliable RC processing difference was observed in this region.

To examine whether older and younger adults differed in their region-by-region RT trajectories, we conducted a growth curve analysis on reading times across regions. This approach was chosen to explore whether the two age groups showed different patterns of change over the course of sentence processing. Region was coded as a positional variable, and orthogonal polynomial terms were used to capture both the linear trend and the quadratic trend. We fitted a linear mixed-effects model to log-transformed reading times, with group, clause type, and the linear and quadratic region terms as fixed effects and with random intercepts plus random linear and quadratic slopes for subjects and random intercepts for items. Results show that there was a significant main effect of group, with younger adults showing shorter reading times than older adults (*β* = −0.43, *SE* = 0.07, *t* = −5.83, *p* < 0.001). The main effect of clause type was not significant (*β* = 0.01, *SE* = 0.01, *t* = 0.40, *p* = 0.686), indicating that the two types of RCs did not differ significantly in reading times. Both the linear region term (*β* = 8.58, *SE* = 1.58, *t* = 5.44, *p* < 0.001) and the quadratic region term (*β* = 16.70, *SE* = 1.41, *t* = 11.87, *p* < 0.001) were significant, showing that reading times changed systematically across the sentence and that this change was clearly not linear. Critically, the interaction between group and the linear trend was significant (*β* = 5.29, *SE* = 2.32, *t* = 2.28, *p* = 0.025), as was the interaction between group and the quadratic trend (*β* = −5.28, *SE* = 2.07, *t* = −2.56, *p* = 0.012). These interactions indicate that older and younger adults differed in the overall shape of their RT trajectories across sentence regions. As shown in Figure 4, older adults showed greater fluctuation across regions, whereas younger adults showed a flatter pattern. By contrast, the interactions between clause type and the linear trends were not significant (*p*s > 0.10); the three-way interaction between group, clause type, and the trend was not significant (*β* = 1.93, *SE* = 1.63, *t* = 1.19, *p* = 0.236); and the three-way interaction with the quadratic trend was only marginal (*β* = −2.69, *SE* = 1.63, *t* = −1.66, *p* = 0.098). These results suggest that the age difference lies in how processing load is distributed over time as the sentence unfolds. Specifically, older adults showed a larger drop from R1 to R3 and a sharper increase from the later regions to the end of sentences. Younger adults showed the similar pattern, but with smaller region-to-region fluctuations and a smaller sentence-final increase.

## 4. Discussion

The purpose of this study was to explore the differences in processing difficulty between SRCs and ORCs in Mandarin-speaking older adults. A self-paced reading experiment was implemented among a group of younger and older speakers of Mandarin Chinese to explore age differences in RC comprehension. Key findings and their implications are discussed in the following section.

### 4.1. Age Differences in Mandarin RC Processing

Analysis of responses to comprehension questions showed that the older group had significantly lower accuracy in comprehending both types of RCs than the younger group. Furthermore, the older group’s response times were significantly longer than that of the younger group across all sentence segments, indicating that the ability to comprehend relative structures declines with aging, and the older group’s ability to process complex syntactic structures significantly deteriorates. This finding is largely consistent with previous studies from the aging population (Kemper, 1986, 1987; Baum, 1993; Caplan et al., 2011; Liu et al., 2019; Liu & Wang, 2019).

The analysis of comprehension accuracy revealed that Chinese SRCs were more difficult to comprehend than ORCs in both younger and older adults. The effect was significantly stronger in older adults than in younger adults. This processing asymmetry was also found in participants’ response times to comprehension questions. This largely aligns with the prediction of memory-based accounts regarding the difficulty of processing RCs in older adults. Compared to younger adults, older adults are more sensitive to manipulation of syntactic structure, and syntactic complexity has a greater impact on performance in this group. This finding is supported by other similar studies (e.g., Zurif et al., 1995; Stine-Morrow et al., 2000; He et al., 2017). One possible explanation is that the longer linear dependency in SRCs places greater demands on working memory and dependency formation, making thematic role assignment more challenging for older adults.

The analysis of speed-accuracy relationships provides an additional insight into the accuracy patterns. One possible explanation for the stronger effect of RC type is that older adults might adopt a more conservative response strategy, sacrificing speed in order to maintain accuracy. However, our trade-off analysis does not support this account. Although a significant relationship between accuracy and speed was observed across participants, the interaction between response times and age group was not significant. This indicates that the relationship between speed and accuracy was statistically equivalent for younger and older adults. In other words, older adults did not benefit more from slowing down than younger adults. The present study found that longer response times were associated with lower accuracy, which suggests that longer response times did not reflect a deliberate trade-off between speed and accuracy, but rather higher cognitive load and processing difficulty. In addition, we found no significant learning effects or adaptation over time, indicating that the observed differences in accuracy are not simply due to fatigue or changes in task strategies across trials. We found a marginally significant interaction between trial order and group. Although this trend may reflect subtle differences in performance between age groups, the effect did not reach statistical significance. Therefore, the larger SRC disadvantage in the older group is unlikely to be explained by differences in decision strategy or task experience and more likely reflects an age-related limitation in processing syntactically complex structures.

Analysis of RTs for the head noun and main verb revealed that clause type had no significant effect on RTs. No significant differences were found between the older and younger groups in terms of clause type effect either. These results do not provide clear support for either memory-based accounts or experience-based accounts. The results of the present study are more consistent with cue-based retrieval and similarity-based interference accounts, which emphasize the difficulty of retrieving the appropriate representation from memory during sentence processing (Gordon et al., 2001, 2006; Lewis & Vasishth, 2005). According to these accounts, if two noun phrases share similar cues, retrieval becomes more difficult. This may lead to wrong interpretations, even when reading times do not show a clear difference at each region. This might explain the discrepancy between our online and offline results. Older adults could read the sentences at a pace similar to younger adults, but they were less successful in retrieving the correct thematic roles at the end. In this way, interference may show up more clearly in offline comprehension than in online RTs. This idea may be especially relevant for Mandarin Chinese. In Mandarin ORCs, the agent precedes the patient, whereas in SRCs the patient precedes the agent. Due to such differences, ORCs provide clearer cues for thematic-role assignment, while SRCs may create more competition during retrieval. This may be one reason why SRCs were harder for older adults in the present study. The SRC disadvantage here suggests that older adults are more vulnerable to retrieval interference during thematic-role assignment. In some way, an interference-based perspective may help bridge memory-based and experience-based accounts. It retains a central role for memory, because sentence comprehension depends on the retrieval of linguistic representations from memory (Lewis & Vasishth, 2005). At the same time, retrieval-based theories may accommodate frequency-based expectations via base-level activation (Parker et al., 2017). In this sense, interference-based accounts provide a useful framework for integrating these two approaches.

Although cue-based retrieval and similarity-based interference accounts offer a plausible explanation for our findings, other factors may also have weakened RC-type differences during incremental processing. We therefore consider several other factors that may have reduced RC-type and age effects in the RT data. First, this may be partly attributed to the temporary syntactic ambiguity in Chinese RCs. As left-branching structures, Chinese RCs place both the relativizer *de* and the head noun at the right edge of the clause. In ORCs, *de* can also function as a post-nominal genitive marker (Jäger et al., 2015; Zhou et al., 2018), creating a temporary ambiguity that is not resolved until the main clause verb. Comprehenders may analyze the RC as a verbal phrase at first and revise their interpretation at the point of disambiguation. In principle, this ambiguity could increase processing difficulty. However, its effect does not necessarily lead to an ORC disadvantage, as online processing in Mandarin RCs involves many factors, including structural integration demands and the point at which disambiguating information becomes available. The processing cost at the subject and main verb regions may reflect the effort in ambiguity resolution as well as RC processing difficulty. Temporary ambiguity may have masked the RC effect in the online data. Our findings are not consistent with prior studies that reported RC asymmetry. This divergence might be attributed to differences in the design of experimental stimuli. Earlier self-paced reading research has reported online RC differences in Mandarin (F. Hsiao & Gibson, 2003), whereas subsequent studies have argued that such effects are highly sensitive to local ambiguity in experimental stimuli (Gibson & Wu, 2013; Jäger et al., 2015). In particular, Jäger et al. (2015) found that when local ambiguity was controlled, both self-paced reading and eye-tracking could reveal clearer online contrasts.

The lack of an RC effect in online processing might also be related to the nature of cognitive resources involved in sentence comprehension. Caplan and Waters (1999)’s separate-sentence-interpretation-resource (SSIR) theory posits that online syntactic processing relies on a specialized, domain-specific working memory system that is independent of general cognitive resources. They argue that online interpretive processes are distinct from post-interpretive processes, and the two processes tap into different types of memory resources. While domain-general working memory is responsible for post-interpretive processes, such as storing and manipulating information, interpretive processes rely on a system dedicated to syntactic processing. Accordingly, differences in RC type are more likely to emerge in comprehension outcomes than in process. This framework, therefore, helps to explain why an RC effect was found in offline comprehension accuracy but not in the online data. If domain-general working memory is not recruited in online syntactic processing, age-related declines in general working memory capacity would not necessarily lead to differences in online reading times. This pattern is supported by empirical studies showing that age-related decline in working memory capacity affects the later stages of sentence processing, but online processing remained largely intact (Caplan et al., 2011; G. Waters & Caplan, 2005). The finding seems to suggest that the specialized verbal resources may not be compromised by biological aging. This is echoed by neuroimaging studies, which demonstrate that syntactic processing is resilient to aging and the core frontotemporal network for syntax remains actively engaged in syntactic processing tasks (Campbell et al., 2016; Tyler et al., 2010). However, future studies are needed to provide direct evidence in support of this argument.

Notably, no reliable RC difference was observed not only in older adults but also in the younger group in this study. This pattern suggests that the absence of an RC effect is not specific to aging. It is possible that the processing cost difference between SRCs and ORCs in these regions was simply too small to reach the threshold to produce significant differences. RT measures are noisy and may lack the sensitivity to detect subtle syntactic effects, especially when multiple sources of variability such as ambiguity resolution are present. Therefore, the results should not be interpreted as evidence against structural processing asymmetries. The present sample size may also limit the sensitivity to detect subtle effects. However, the consistency of the null effect across multiple regions and analyses suggests that, within the current design, any RC effect is likely to be weak or context-dependent. Future research could refine the experimental design by introducing additional task manipulations, such as increasing the frequency of disambiguating cues, and by increasing sample size. Moreover, using eye-tracking methods could allow for more in-depth analysis of processing difficulty across smaller time windows, providing a clearer picture of how different syntactic structures are processed across groups.

To explore how aging influences the processing effort across sentences, we performed a growth curve analysis of reading times. Interestingly, older adults showed a trajectory with greater curvature and larger region-to-region modulation. Reading times dropped more sharply into the middle portion of the sentence and then rose toward the sentence end. Younger adults followed the same trajectory, but the changes were smaller and more evenly distributed. Thus, the age difference appears to lie in the processing trajectory. This pattern might be explained by a resource-based account. Reduced online processing efficiency may make it harder for older adults to distribute integration effort evenly across regions. By contrast, the flatter trajectory in younger adults reflects a stronger ability to maintain materials in memory and defer integration until later parts of the sentence become visible.

### 4.2. Theoretical Implications

The results of this study offer important insights into existing theories on sentence processing. Compared with the experience-based prediction, the present findings are more compatible with resource-sensitive accounts and with a cue-based retrieval explanation. However, some studies have questioned the explanatory power of the dependency locality theory for the processing of Chinese RCs (Wu, 2012). The discrepancy between the present findings and those of some previous studies may be attributed to differences in participant populations. Given the specific cognitive characteristics of older participants, memory-based accounts may be more relevant for understanding the performance in this sample. Previous studies have found that the difficulties in sentence processing by older adults are usually caused by insufficient working memory resources (Stine, 1995; Zurif et al., 1995). Due to memory constraints, older adults are more sensitive to changes in the cognitive load of language structures. Although they have richer language experience, working memory decline still has a negative effect on language processing ability. This study suggests from the perspective of aging that memory resources play a crucial role in sentence processing. This view is supported by research on English RC processing in older adults (Stine-Morrow et al., 2000; Zurif et al., 1995). Stine-Morrow et al. (2000) compared the differences in processing English RCs between older adults and young adults and found that older adults could not allocate cognitive resources as effectively as young adults when processing the cognitively more demanding ORCs. Working memory decline is the main reason for the decline in the effectiveness of resource allocation in RC processing in older adults. Stine (1995) also pointed out that the decline in language processing ability is closely related to the decline in working memory in older adults, as older adults have difficulty meeting the resource requirements for constructing meaningful representations of texts. These studies demonstrate from the perspective of aging that sentence processing is a complex computational process that consumes cognitive resources.

Current processing theories often assume an ideal processor with sufficient resources, but this assumption may be less appropriate for aging populations. We argue that sentence processing is a process involving both resource demand and availability. Comprehenders’ performance is determined by the degree of match between the computational demands imposed by the sentence and the cognitive resources available to the processor. Experience-based accounts posit that structures with higher frequency are easier to process. However, frequency-based advantages can be reduced under resource constraints. Processing difficulty is dynamic and varies across individuals and tasks. The assumption of an ideal processor does not seem to reflect the reality of language processing in the aging population. A comprehensive framework should recognize that processing difficulty is not only determined by the characteristics of linguistic structures such as frequency or syntactic complexity but also by the capacity of the cognitive system. We suggest that future theories of sentence processing focus more on the interaction between processing demands and resource availability to give a more ecologically valid account of language processing across the lifespan.

Finally, it should be noted that our findings do not imply that experience-based theories are invalid. Rather, different theoretical accounts may apply under different conditions. As our study did not include corpus-based evidence, it cannot directly test the role of distributional experience in Mandarin RC processing. It therefore remains possible that experience-based factors contribute to RC processing under some conditions. Experience-based effects are more likely to emerge when processing resources are sufficient and structural demands are relatively low. Under resource-limited conditions, however, such as in aging, the influence of frequency may be overridden by resource constraints. From this perspective, memory-based and experience-based accounts should not be viewed as mutually exclusive but as complementary components of a unified framework in which processing outcomes reflect the interaction between probability and the cognitive demand of linguistic structures.

## 5. Conclusions

The present study employed a self-paced reading paradigm to examine the processing of Mandarin RCs in different age groups. The results revealed that SRCs were more difficult than ORCs in both younger and older adults, and this asymmetry was exacerbated in older adults. The observed age-related differences in comprehension accuracy cannot be attributed to strategic trade-offs between speed and accuracy. With respect to online processing, older adults showed generally slower performance than younger adults, reflecting an overall age-related slowing. However, no significant RC effects or group-by-RC interactions were found in reading times. Normal aging primarily affects sentence comprehension at later stages involving integration and decision-making, whereas online syntactic parsing remains relatively intact. The findings further indicate that changes in general cognitive resources impose important constraints on age-related variation in language performance.

However, it should be acknowledged that this study is not without limitations. First, this study includes a relatively small number of experimental items. With 30 item sets, the study may have had limited sensitivity to detect subtle online processing differences. Future research with a larger item set would help determine whether the present reading-time patterns remain stable. Moreover, older adults often have reduced motor control, which may lead to delayed or imprecise keypress responses during experimental tasks. Such motor-related delays may introduce systematic bias into RT measures. As a result, we should be cautious to interpret RTs as a sole index of sentence processing efficiency in this population. The present findings from reading times should be viewed as tentative. Electrophysiological or neuroimaging studies are needed to further explore the age-related changes in the online processing of Mandarin RCs.

## Figures and Tables

**Table 1:** Mean RTs by RC type and age group (ms).

Region	Older ORC	Older SRC	Younger ORC	Younger SRC
Region 1	775 (432)	866 (507)	399 (150)	399 (176)
Region 2	613 (307)	633 (332)	396 (162)	403 (168)
Region 3	530 (226)	546 (266)	379 (140)	385 (131)
Region 4	608 (314)	611 (310)	408 (209)	424 (199)
Region 5	731 (404)	715 (371)	451 (211)	474 (261)
Region 6	1074 (722)	1154 (897)	788 (705)	791 (701)

## Data Availability

The data presented in this study are available on request from the author.

## Appendix A

**Table A1:** Linear mixed-effects results for reading times at the head noun.

Region	Predictor	Estimate	*SE*	*t*	*p*
Head noun	(Intercept)	6.32	0.06	113.57	<0.05
Head noun	Group	−0.39	0.08	−4.82	<0.05
Head noun	Type	0.001	0.02	0.04	0.965
Head noun	Group × Type	0.02	0.04	0.67	0.502
